# The Calcium Phosphate Matrix of FGF-2-Apatite Composite Layers Contributes to Their Biological Effects

**DOI:** 10.3390/ijms150610252

**Published:** 2014-06-10

**Authors:** Hirotaka Mutsuzaki, Atsuo Ito, Yu Sogo, Masataka Sakane, Ayako Oyane, Masashi Yamazaki

**Affiliations:** 1 Department of Orthopaedic Surgery, Ibaraki Prefectural University of Health Sciences, 4669-2 Ami Ami-machi, Inashiki-gun, Ibaraki 300-0394, Japan; E-Mail: mutsuzaki@ipu.ac.jp; 2 Human Technology Research Institute, National Institute of Advanced Industrial Science and Technology (AIST), Central 6, 1-1-1 Higashi, Tsukuba, Ibaraki 305-8566, Japan; E-Mail: yu-sogou@aist.go.jp; 3 Department of Orthopaedic Surgery, University of Tsukuba, 1-1-1 Tennodai, Tsukuba, Ibaraki 305-8575, Japan; E-Mails: sakane-m@md.tsukuba.ac.jp (M.S.); masashiy@md.tsukuba.ac.jp (M.Y.); 4 Nanosystem Research Institute, National Institute of Advanced Industrial Science and Technology (AIST), Central 4, 1-1-1 Higashi, Tsukuba, Ibaraki 305-8562, Japan; E-Mail: a-oyane@aist.go.jp

**Keywords:** fibroblast growth factor 2, apatite coating, pin tract infection, external fixation, fixation strength, Ca/P molar ratio

## Abstract

The purpose of the present study was to fabricate fibroblast growth factor (FGF)-2-apatite composite layers on titanium (Ti) pins in one step at 25 °C using a supersaturated calcium phosphate (CaP) solution, and to evaluate the physicochemical characteristics and biological effects of the coated Ti pins compared with coated Ti pins fabricated at 37 °C. Ti pins were immersed in a supersaturated CaP solution containing 0.5, 1.0, or 2.0 µg/mL FGF-2 at 25 °C for 24 h (25F0.5, 25F1.0, and 25F2.0) or containing 4.0 µg/mL FGF-2 at 37 °C for 48 h (37F4.0). Except for the 25F0.5, the chemical compositions and the mitogenic activity levels of FGF-2 of the composite layers formed by these two methods were similar, except for the Ca/P molar ratio, which was markedly smaller at 25 °C (1.55–1.56 ± 0.01–0.02, *p* = 0.0008–0.0045) than at 37 °C (1.67 ± 0.11). Thus, either the apatite was less mature or the amount of amorphous calcium phosphate was higher in the composite layer formed at 25 °C. *In vivo*, the pin tract infection rate by visual inspection for 37F4.0 (45%) was lower than that for 25F1.0 (80%, *p* = 0.0213), and the rate of osteomyelitis for 37F4.0 (35%) was lower than that for 25F0.5 (75%, *p* = 0.0341). The extraction torque for 37F4.0 (0.276 ± 0.117 Nm) was higher than that for 25F0.5 (0.192 ± 0.117 Nm, *p* = 0.0142) and that for 25F1.0 (0.176 ± 0.133 Nm, *p* = 0.0079). The invasion rate of *S. aureus* for 37F4.0 (35%) was lower than that for 25F0.5 (75%, *p* = 0.0110). On the whole, the FGF-2-apatite composite layer formed at 25 °C tended to be less effective at improving fixation strength in the bone-pin interface and resisting pin tract infections. These results suggest that the chemistry of the calcium phosphate matrix that embeds FGF-2, in addition to FGF-2 content and activity, has a significant impact on composite infection resistance and fixation strength.

## 1. Introduction

Pin tract infections and loose anchorages are severe problems that can occur during external fixation [1,2,3,4,5,6,7]. Pin tract infections can induce loose anchorages when affecting bone, and the loose anchorages can also exacerbate pin tract infections. Therefore, preventing pin tract infections is essential for the successful external fixation of bone. Conventionally, pin tract infections are prevented by intensive pin site care [8]. More recently, modifications of pin surfaces to prevent pin tract infections have been developed, including hydroxyapatite coatings for increasing fixation strength in the bone-pin interface [9,10], as well as a titanium-copper alloy [11], silver coatings [12,13], a titanium oxide photocatalyst coating [14], and antimicrobial-loaded hydroxyapatite [15] and polymer coatings [16].

Accelerating tissue regeneration around the pin tract is another approach to control bacterial infection. In a previous study, we formed fibroblast growth factor (FGF)-2-apatite composite layers on titanium (Ti) pins by immersing them in an infusion fluid-based supersaturated calcium phosphate (CaP) solution containing FGF-2 at 37 °C for 48 h [17]. FGF-2 is a growth factor that promotes dermis formation [18], bone formation [19], and angiogenesis [20]. The FGF-2-apatite composite layer effectively prevented pin tract infections [17]. The improved resistance to pin tract infections was attributed to the improved bone-pin interface strength and the accelerated wound healing that was associated with the formation of Sharpey’s fiber-like tissue and accompanying angiogenesis around the pin tract [17,21].

Reducing the fabrication temperature and time of the FGF-2-apatite composite layers to enhance their biological activity and practicality is an important challenge. The previous 37 °C and 48 h fabrication temperature and time, respectively, are associated with a significant risk of losing the FGF-2 biological activity [22,23]. Although lowering the fabrication temperature is favorable for retaining FGF-2 biological activity, lower fabrication temperatures inhibit nucleation and growth of apatite due to its retrograde solubility. Therefore, the fabrication of FGF-2-apatite composite layers at room temperature previously required a two-step method in which the apatite layers are first coated in a supersaturated CaP solution containing no FGF-2 at 37 °C, and then overlaid with an FGF-2-apatite composite layer in another supersaturated CaP solution containing FGF-2 at 25 °C [23]. Recently, we succeeded in coating apatite on Ti pins using a one-step process at 25 °C by increasing the concentrations of calcium (2.43-fold) and phosphate (1.62-fold) ions compared with the solution concentrations used at 37 °C [24]. An equivalent increase in calcium and phosphate ion concentrations is likely to be effective for fabricating FGF-2-apatite composite layers in one step at 25 °C.

The purpose of the present study was (1) to fabricate FGF-2-apatite composite layers on Ti pins in one step at 25 °C using an infusion fluid-based supersaturated CaP solution containing different concentrations of FGF-2; and (2) to evaluate the physicochemical characteristics, biological activity, resistance to pin tract infection, and bone fixation strength of the coated Ti pins compared with Ti pins coated at 37 °C.

## 2. Results and Disscussion

### 2.1. Characterization of the FGF-2-Apatite Composite Layers

**Figure 1:**
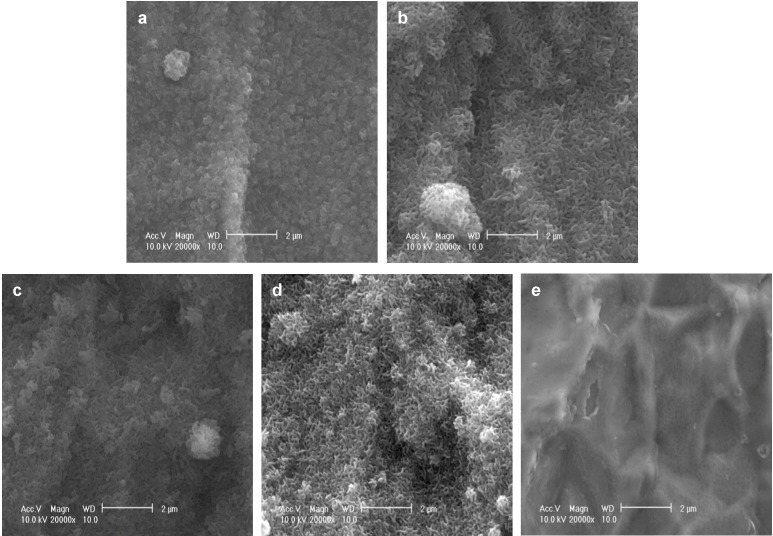
SEM (scanning electron microscopy) images of the surfaces of (**a**) 37F4.0; (**b**) 25F0.5; (**c**) 25F1.0; (**d**) 25F2.0; and (**e**) Uncoated Ti pin.

As reported previously, after immersion in a supersaturated CaP solution containing 4.0 µg/mL FGF-2 at 37 °C for 2 days (37F4.0), Ti pins were entirely and homogeneously coated with a fine-structured layer, as confirmed by scanning electron microscopy (SEM) (Figure 1a). Similar fine-structured layers were also observed on Ti pins after immersion in a supersaturated CaP solution containing 2.43-fold higher calcium and 1.62-fold higher phosphate ion concentrations with 0.5, 1.0, or 2.0 µg/mL FGF-2 at 25 °C for 1 day (25F0.5, Figure 1b; 25F1.0, Figure 1c; or 25F2.0, Figure 1d). The layer for the 37F4.0 appeared to be slightly denser than that for the 25F0.5, 25F1.0, and 25F2.0. No such layers were observed on the uncoated Ti pins (UN, Figure 1e).

**Figure 2:**
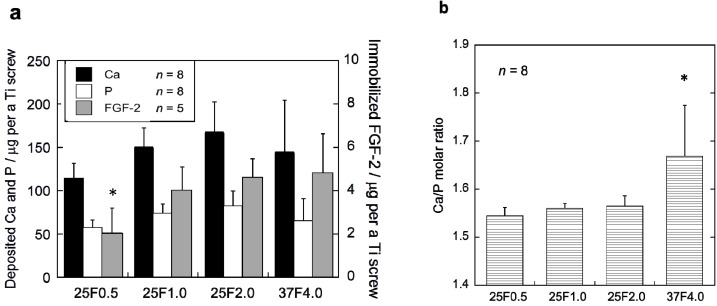
(**a**) Ca, P and FGF-2 contents; and (**b**) the Ca/P molar ratios of the composite layers in the 25F0.5, 25F1.0, 25F2.0, and 37F4.0 groups. * *p* < 0.05 compared with 25F2.0 and 37F4.0 groups in (**a**); and compared with the 25F0.5, 25F1.0, and 25 F2.0 groups in (**b**).

The FGF-2-apatite composite layers on the Ti pins coated at 25 °C for 1 day contained similar FGF-2, Ca, and P contents to those coated at 37 °C for 2 days, but the Ca/P molar ratios were significantly different. The FGF-2 content in the composite layers was quantified using the Bradford method (Figure 2a). The FGF-2 content of the 37F4.0 (4.72 ± 1.91 µg), 25F2.0 (4.62 ± 0.86 µg), and 25F1.0 (3.97 ± 1.14 µg) groups were not significantly different. Only the FGF-2 content of the 25F0.5 (2.04 ± 1.18 µg) group was significantly lower than the 25F2.0 (*p* = 0.0221) and 37F4.0 (*p* =0.0229) groups. No significant differences were observed in Ca or P contents among all the groups. However, the layer Ca/P molar ratios of the 25F0.5 (1.55 ± 0.02, *p* = 0.0008), 25F1.0 (1.56 ± 0.01, *p* = 0.0024), and 25F2.0 (1.56 ± 0.02, *p* = 0.0045) groups were significantly lower than that of the 37F4.0 group (1.67 ± 0.11) (Figure 2b).

**Figure 3:**
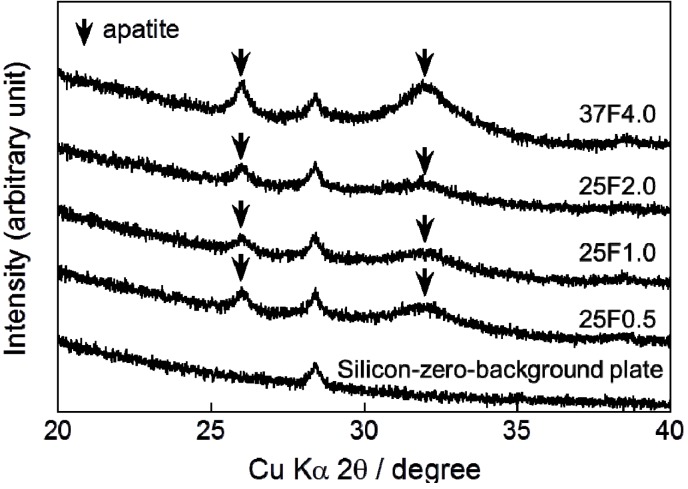
XRD (X-ray diffraction) patterns from a silicon-zero-background plate and CaP (calcium phosphate) deposited on the 25F0.5, 25F1.0, 25F2.0, and 37F4.0 groups. Arrows indicate the diffraction peaks corresponding to those for apatite.

Very broad and small peaks appeared in the X-ray diffraction (XRD) patterns of all the composite layers at approximately 26° and 32° (Figure 3). These peaks confirmed that the main crystalline phase of the layers was low-crystalline apatite.

**Figure 4:**
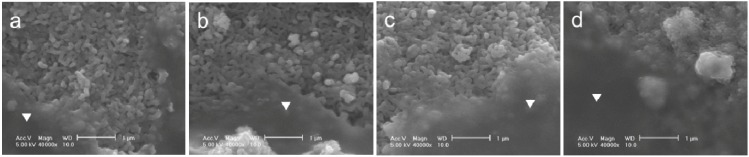
SEM images of the FGF-2-apatite composite layers after the Scotch^®^ tape detachment test on (**a**) 25F0.5; (**b**) 25F1.0; (**c**) 25F2.0; and (**d**) 37F4.0. Glue on a smooth surface is marked with an inverted triangle.

The adhesion strength of the FGF-2-apatite composite layers on Ti pins coated at 25 °C for 1 day was comparable to that of the layers coated at 37 °C for 2 days. After a Scotch^®^ tape detachment test, the surfaces of the 25F0.5, 25F1.0, 25F2.0, and 37F4.0 pins remained covered with the FGF-2-apatite composite layers, and glue was observed on the surfaces in all the samples (Figure 4). Thus, the composite layers adhered to the Ti pins so strongly that glue from the Scotch^®^ tape remained on the composite layer without detaching the layer from the Ti pin.

**Figure 5:**
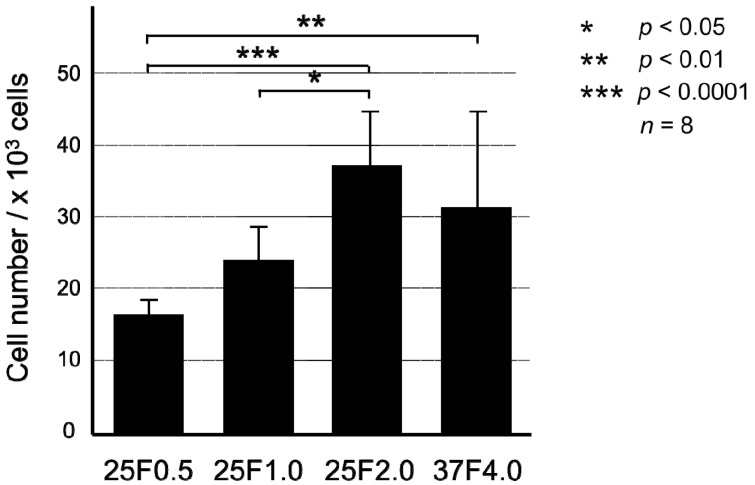
Number of NIH3T3 cells extracted with citric acid buffer after culture for 72 h in serum-free medium from the FGF-2-apatite composite layers of the 25F0.5, 25F1.0, 25F2.0, and 37F4.0 groups.

The biological activity of the FGF-2-apatite composite layers on Ti pins coated at 25 °C for 1 day was comparable to that of those coated at 37 °C for 2 days, except for the 25F0.5 group (Figure 5). The mitogenic activity of the immobilized FGF-2, as determined by the proliferation of NIH3T3 cells cultured in serum-free medium, increased with increasing FGF-2 concentration added to the supersaturated CaP solution for the 25 °C groups. There were no significant differences in mitogenic activity between the 37F4.0 and 25F1.0 (*p* = 0.2897) groups, or between the 37F4.0 and 25F2.0 (*p* = 0.5373) groups.

### 2.2. Classification of Pin Tract Infections by Visual Inspection

**Figure 6:**
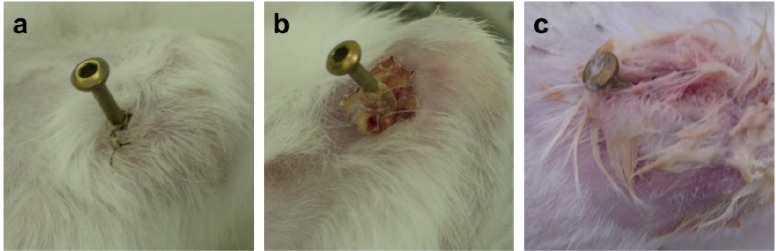
Typical appearances of pin tract infections 4 weeks after the operation. (**a**) Grade 0v: No redness, discharge, or pin loosening; (**b**) Grade 1v: Redness and discharge around the pin, but no pin loosening; and (**c**) Grade 2v: Redness and discharge around the pin with pin loosening due to osteomyelitis.

**Figure 7:**
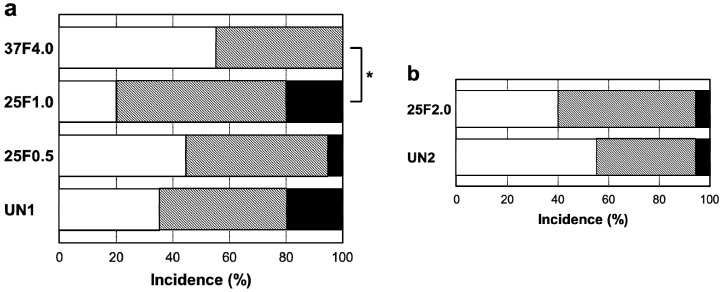
Percent incidence of Grades 0v, 1v, and 2v pin tract infections determined by visual inspection of the (**a**) UN1, 25F0.5, 25F1.0, and 37F4.0 groups; and for the (**b**) UN2 and 25F2.0 groups. 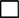: Grade 0v, 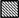: Grade 1v, 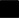: Grade 2v. * *p* < 0.05.

Surprisingly, the FGF-2-apatite composite layers on Ti pins fabricated at 25 °C for 1 day failed to show an improved resistance to pin tract infections compared with those fabricated at 37 °C for 2 days. The typical appearances of Grades 0v (no redness), 1v (skin infection without pin loosening), and 2v (infection with pin loosening) after visual inspection of pin tract infections are shown in Figure 6. The incidence rates of Grade 0v were 55% (37F4.0, *n* = 11/20), 20% (25F1.0, *n* = 4/20), 45% (25F0.5, *n* = 9/20), and 35% (UN1, *n* = 7/20) (Figure 7a). The rates of Grade 1v were similar among all the groups, ranging from 45% to 60%. The rates of Grade 2v were 0% (37F4.0, *n* = 0/20), 20% (25F1.0, *n* = 4/20), 5% (25F0.5, *n* = 1/20), and 20% (UN1, *n* = 4/20). The resistance to pin tract infection (Grade 0v:1v:2v ratio) for the 25F1.0 group was significantly lower than that of the 37F4.0 group (*p* = 0.0213). It is worth noting that the *p* value for the difference in Grade 0v:1v:2v ratio between the 37F4.0 and UN1 groups was markedly lower (*p* = 0.0868) than that between the 25F1.0 and UN groups (*p* = 0.5361), and between 25F0.5 and UN groups (*p* = 0.3494). This suggests that there was no improvement in resistance to pin tract infection for the 25 °C groups compared with the UN1 group (uncoated Ti pins). We carried out another set of animal experiments to compare the 25F2.0 and UN2 groups. The 25F2.0 group showed no improvement in resistance to pin tract infection compared with UN2 group (another uncoated Ti pins) (Figure 7b).

### 2.3. Classification of Inflammation Grade by Histological Observation

**Figure 8:**
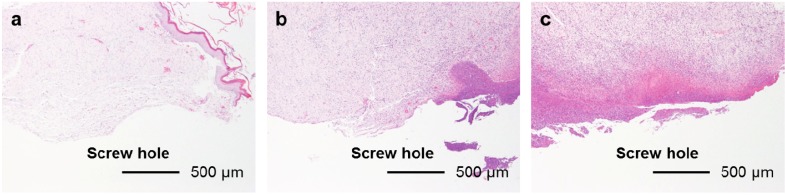
Typical histological appearances of soft tissue inflammation along the pin tract 4 weeks after the operation. (**a**) Grade 0s: No inflammation; (**b**) Grade 1s: Partial inflammation; and (**c**) Grade 2s: Severe inflammation.

**Figure 9:**
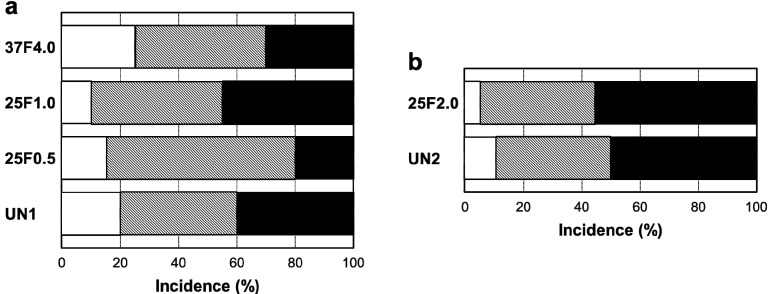
Percent incidence of Grades 0s, 1s, and 2s pin tract infections determined by histological observation of (**a**) the UN1, 25F0.5, 25F1.0, and 37F4.0 groups; (**b**) the UN2 and 25F2.0 groups. 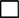: Grade 0s, 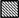: Grade 1s, 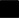: Grade 2s.

The FGF-2-apatite composite layers on Ti pins fabricated at 25 °C for 1 day showed similar levels of soft tissue inflammation as those fabricated at 37 °C for 2 days. The typical histological appearances for Grades 0s (no inflammation), 1s (partial inflammation), and 2s (severe inflammation) soft tissue inflammation along the pin tract are shown in Figure 8. The percent incidence of inflammation in the UN1, 25F0.5, 25F1.0, and 37F4.0 groups ranged from 10% to 20% (Grade 0s), 40% to 65% (Grade 1s), and 20% to 45% (Grade 2s). No significant differences were found in the Grade 0s:1s:2s ratios between any pair of the UN1, 25F0.5, 25F1.0, and 37F4.0 groups (*p* = 0.2405–0.7962) (Figure 9a). Similarly, no significant difference was found in the Grade 0s:1s:2s ratio between the UN2 and 25F2.0 groups (*p* = 0.5957) (Figure 9b).

**Figure 10:**
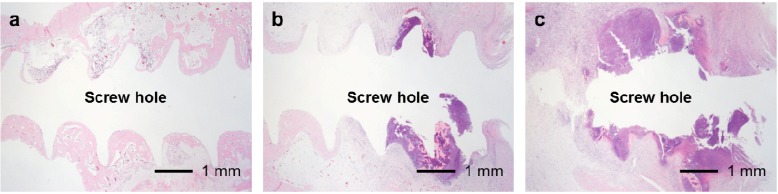
Typical histological appearances of inflammation in the bone tissue along the pin tract 4 weeks after the operation. (**a**) Grade 0b: No osteomyelitis; (**b**) Grade 1b: Partial osteomyelitis; and (**c**) Grade 2b: Severe osteomyelitis.

**Figure 11:**
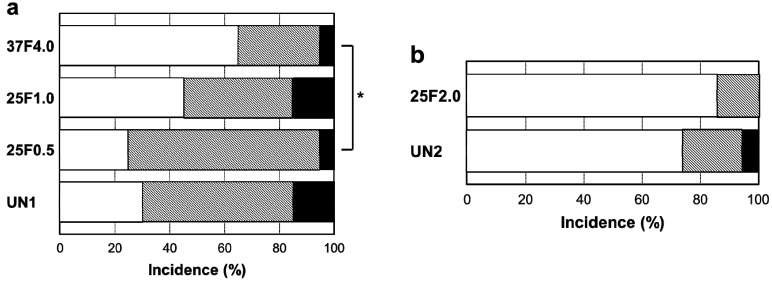
Percent incidence of Grades 0b, 1b, and 2b osteomyelitis determined by histological observation of (**a**) the UN1, 25F0.5, 25F1.0, and 37F4.0 groups; (**b**) the UN2 and 25F2.0 groups. 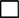: Grade 0b, 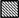: Grade 1b, 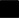: Grade 2b. * *p* < 0.05.

The FGF-2-apatite composite layers on Ti pins coated at 25 °C for 1 day failed to reduce inflammation in the bone tissue compared with those coated at 37 °C for 2 days. The typical histological appearances for Grades 0b (no osteomyelitis), 1b (partial osteomyelitis), and 2b (severe osteomyelitis) inflammation in bone tissue along the pin tract are shown in Figure 10. The incidence rates of Grade 0b were 65% (37F4.0, *n* = 13/20), 45% (25F1.0, *n* = 9/20), 25% (25F0.5, *n* = 5/20), and 30% (UN1, *n* = 6/20) (Figure 11a). The incidence rates of Grade 1b were 30% (37F4.0, *n* = 6/20), 40% (25F1.0, *n* = 8/20), 70% (25F0.5, *n* = 14/20), and 55% (UN1, *n* = 11). The rates of Grade 2b were nearly the same among all groups, varying from 5% to 15%. The resistance to pin tract infection in bone tissue (Grade 0b:1b:2b ratio) of the 25F0.5 group was significantly lower than that of the 37F4.0 group (*p* = 0.0341). It is worth noting that the *p* value for the difference in Grade 0b:1b:2b ratios between the 37F4.0 and UN1 groups was markedly lower (*p* = 0.0801) than that between the 25F1.0 and UN1 groups (*p* = 0.5846), and between the 25F0.5 and UN1 groups (*p* = 0.4841), which suggests that there was no improvement in resistance to pin tract infection in bone tissue for the 25 °C groups compared with the UN1 group. In the second set of animal experiments, the 25F2.0 group showed no improvement in resistance to pin tract infection in bone tissue (Grade 0b:1b:2b ratio) compared with the UN2 group (*p* = 0.6225) (Figure 11b).

**Figure 12:**
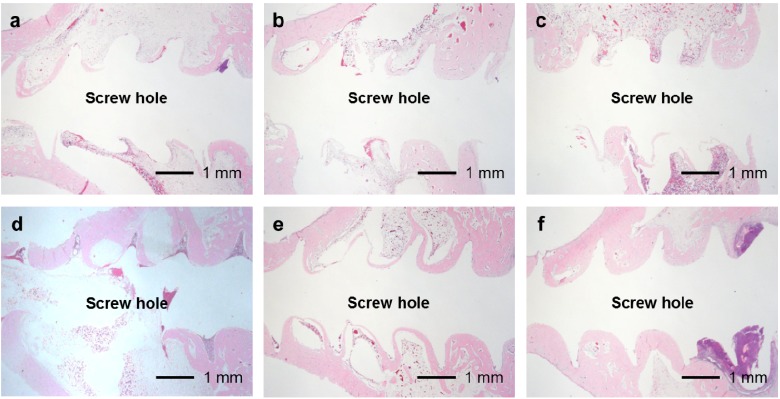
Histological sections showing bone formation in the cortical and medullary cavity regions. No or very little bone formed in the medullary cavity regions in the (**a**) UN1; (**b)** 25F0.5; (**c**) 25F1.0; and (**d**) 25F2.0 groups in the Grade 0b (no osteomyelitis) samples. On the contrary, dense bone formed in the medullary cavity regions in the 37F4.0 group in (**e**) the Grade 0b and in (**f**) the Grade 1b (partial osteomyelitis) samples.

In the UN, 25F0.5, 25F1.0, and 25F2.0 groups, no or very little bone formed in the medullary cavity region in the Grades 0b and 1b osteomyelitis samples. However, in the 37F4.0 group, a considerable amount of dense bone formed in the cortical and medullary cavity regions in the Grades 0b and 1b osteomyelitis samples (Figure 12).

### 2.4. Bacterial Identification in Pin Tracts

**Figure 13:**
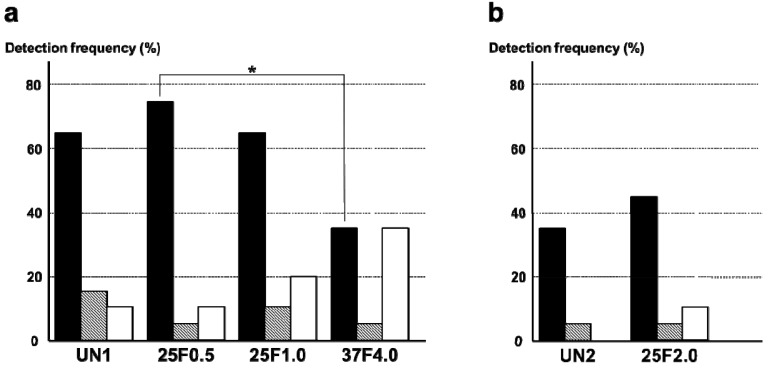
Detection frequencies of *S. aureus* and *E. coli* around the pin tracts of (**a**) the UN1, 25F0.5, 25F1.0, and 37F4.0 groups; (**b**) the UN2 and 25F2.0 groups. 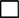: No bacteria, 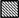: *E. coli*, 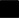: *S. aureus*. * *p* < 0.05.

The FGF-2-apatite composite layers on Ti pins fabricated at 25 °C for 1 day were associated with higher frequencies of *S. aureus* detected around the pin tract than the FGF-2-apatite composite layers fabricated at 37 °C for 2 days. The most frequently detected bacterium around the pin tracts was *S. aureus*. The detection frequency of *S. aureus* was significantly lower in the 37F4.0 group (35%) than in the 25F0.5 group (75%, *p* = 0.0110). Although no significant difference was detected, the detection frequency of *S. aureus* in the 37F4.0 group (35%) was also lower than in the UN1 (65%, *p* = 0.0578) and 25F1.0 (65%, *p* = 0.0578) groups. *E. coli* were also detected in all groups. Their detection frequencies ranged from 5% to 15%, and no significant differences were detected among the UN1, 25F0.5, 25F1.0, and 37F4.0 groups (*p* = 0.1489–0.5). The frequency of no bacterial detection tended to be higher for the 37F4.0 group (35%) than the UN1 group (10%, *p* = 0.0583) and 25F0.5 group (10%, *p* = 0.0583), though no significant differences were detected (Figure 13a). In the second set of animal experiments, no clear differences were found in the frequency of bacterial detection around the pin tract in the 25F2.0 group compared with the UN2 group (*p* = 0.1468–1.0) (Figure 13b).

### 2.5. Extraction Torque

**Figure 14:**
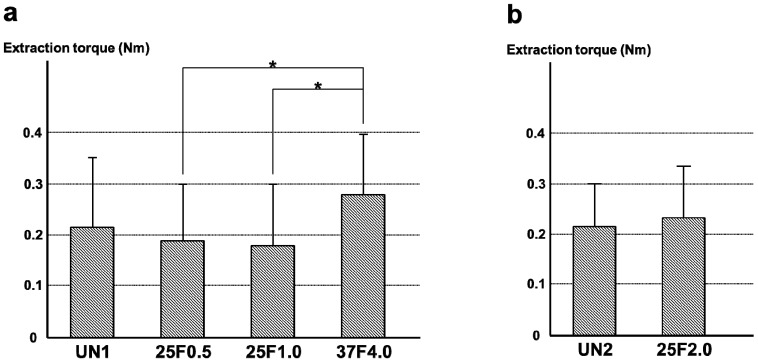
Average values of extraction torque for (**a**) the UN1, 25F0.5, 25F1.0, and 37F4.0 groups; (**b**) the UN2 and 25F2.0 groups. * *p* < 0.05.

The FGF-2-apatite composite layers on Ti pins fabricated at 25 °C for 1 day were associated with lower extraction torque than the FGF-2-apatite composite layers fabricated at 37 °C for 2 days. The average extraction torque for the 37F4.0 group of 0.276 ± 0.117 Nm (*n* = 20), which includes all the osteomyelitis cases, was significantly higher than that of the 25F0.5 group (0.192 ± 0.117 Nm; *n* = 20, *p* = 0.0142) and the 25F1.0 group (0.176 ± 0.133 Nm; *n* = 20, *p* = 0.0079). The average value of extraction torque from all samples in the 37F4.0 group was higher than that of the UN1 group (0.214 ± 0.139 Nm; *n* = 20, *p* = 0.0679), although no significant difference was detected (Figure 14a). The average extraction torque values were not significantly different between the UN2 and 25F2.0 groups (*p* = 0.2793) (Figure 13b).

### 2.6. Discussion

FGF-2-apatite composite layers were successfully fabricated on Ti pins in one step at 25 °C for 1 day using an infusion fluid-based supersaturated CaP solution containing FGF-2. FGF-2 molecule, and CaP crystals and their precursors including CaP clusters can have a positive or a negative surface charge depending on solution chemistry and pH [25,26]. It is expected that FGF-2 interacts with these CaPs when a solution gives opposite electrical charges to FGF-2 and CaPs. Some interaction is also required between Ti and either FGF-2 or the CaPs, or both. The next step would be incorporation of the molecule into the layer by coprecipitation of the molecule and CaPs. Currently, two different mechanisms have been proposed for the process of coprecipitation: one is molecular adsorption followed by crystal nucleation [27], and the other is molecular incorporation into ACP (amorphous calcium phosphate) followed by amorphous-crystalline transformation [28]. As a result, FGF-2-apatite composite layers grow on Ti pins gradually. Previously, one-step fabrication of FGF-2-apatite composite layers was only successful at 37 °C. The reductions in fabrication temperature (from 37 to 25 °C) and time (from 2 days to 1 day) in the present study are significant improvements from the viewpoint of clinical application of external fixation. Kinetics of growth of FGF-2-apatite composite layers on Ti pins at 25 °C is yet to be clarified. As for kinetics of growth of FGF-2-free apatite layers on Ti pins under the same condition, growth and evolution of FGF-2-free apatite layers completes between 12 and 24 h, which was revealed by SEM, XRD, energy dispersive X-ray analysis and chemical analysis by inductively coupled plasma (ICP) spectroscopy (data not shown).

Although a correlation is estimated between the FGF-2 concentration of supersaturated CaP solution, and the amounts of FGF-2, Ca and P deposited in the case of 25 °C groups (Figure 1), the correlation was not statistically significant in the present study; on the contrary, an increased FGF-2 concentration of supersaturated CaP solution even inhibited deposition of Ca and P at 37 °C in the previous study [17]. It is expected that FGF-2, CaP crystals and/or its precursors can have a positive or a negative surface charge in a solution depending on solution composition and pH.

On the whole, however, the FGF-2-apatite composite layers fabricated at 25 °C for 1 day (groups 25F0.5, 25F1.0, and 25F2.0) were less effective at preventing pin tract infections and loose anchorages than those fabricated at 37 °C for 2 days (37F4.0 group), though these two types of layers had similar microstructure, FGF-2 content, adhesion strength, and characteristics of biological activity of FGF-2. The FGF-2-apatite composite layers fabricated at 37 °C for 2 days demonstrated a preventive effect on pin tract infections, as reported previously [17]. We intended for the FGF-2-apatite composite layers fabricated at 25 °C to contain a similar amount of FGF-2 as those fabricated at 37 °C because FGF-2 shows a bell-shaped dose response curve in which both deficient and excessive doses elicit a lower response [29,30]. While the FGF-2 content (Figure 2a) and biological activity of the layers fabricated at 25 and 37 °C (Figure 5) were indeed similar, the composite layers fabricated at 25 °C were less effective at preventing pin tract infections and loose anchorages than those fabricated at 37 °C. This decreased prevention of pin tract infections was evidenced by visual inspection (Figure 7), histological inspection at the bone tissue (Figure 11), and bacterial identification (Figure 13).

The differences in pin tract infection prevention between the FGF-2-apatite composite layers fabricated at 25 and 37 °C may be attributed to differences in the CaP matrix that embeds the FGF-2. In general, CaP matrices of signal molecule-CaP composite layers fabricated by similar processes include low-crystalline apatite [17,31,32], amorphous calcium phosphate (ACP) [33], octacalcium phosphate (OCP), and/or an OCP-like phase [34]. The theoretical Ca/P molar ratios are 1.67 for apatite, 1.50 for ACP, and 1.33 for OCP. In the present study, the Ca/P molar ratio of the FGF-2-apatite composite layers fabricated at 25 °C was 1.55–1.56 and at 37 °C was 1.67 ± 0.11 (Figure 2b). Thus, the molar ratio of the FGF-2-apatite composite layers fabricated at 25 °C corresponds more or less to ACP or contains ACP, while that of those fabricated at 37 °C corresponds to apatite. Because ACP is an amorphous phase that is only weakly detected by XRD, the XRD study (Figure 3) does not rule out the presence of ACP in the layer. ACP is more soluble and less stable than apatite in the physiological environment. This difference in CaP matrix stability may affect the release rate of FGF-2, leading to differences in tissue regeneration due to the dose-dependent FGF-2 response, and eventually altering the prevention of pin tract infections. In other words, the change in Ca/P molar ratio caused a change in optimum FGF-2 content of the layer. It is known that elevated doses of FGF-2 can inhibit bone formation [29]. The differences in CaP matrix stability also can affect bone apposition through differences in the matrix resorption rate. No matter what the controlling mechanism is, the difference in CaP matrix stability may explain the observed result that while some amount of dense bone formed in the cortical and medullary cavity regions in the 37F4.0 group even in the presence of partial osteomyelitis, no or very little bone formed in the medullary cavity regions in the 25F0.5 and 25F1.0 groups (Figure 12). The less bone that forms, the higher the risk of infection, caused by loose anchorages, becomes.

Two limitations of the present study are that the animal experiments were performed in two sets and different analytical methods were used to determine the Ca/P molar ratios in the present and previously reported 37F4.0 groups. The two different sets of animal experiments make direct comparisons between the 25F2.0 group and other groups difficult. The previously reported method for determining Ca/P molar ratios was X-ray photoelectron spectrometry (XPS) [17], which is different than the ICP spectroscopy used in the present study. Because the ICP technique is more accurate than the XPS technique, ICP was used here. The previously reported Ca/P molar ratio for the 37F4.0 group measured by XPS was 1.54 ± 0.11 (*n* = 3) [17], while a value of 1.67 ± 0.11 (*n* = 8) was obtained by the ICP technique in the present study.

Further optimization of the fabrication parameters is needed to reduce the bacterial infection rate and improve the fixation strength at the bone-pin interface for the FGF-2-apatite composite layers prepared at 25 °C. We plan to explore for another fabrication condition under which FGF-2-apatite composite layers with a Ca/P molar ratio at around 1.67 and having higher chemical stability is fabricated at 25 °C, and to carry out comparative studies on FGF-2 release, biological activity of FGF-2, *in vivo* efficacy in preventing pin tract infection, and dissolution and resorption of the layers in order to unambiguously clarify the effect of CaP matrix stability. The fabrication conditions to be optimized include solution compositions, and the FGF-2 content of the supersaturated CaP solution. Theoretically, solution compositions that have a higher Ca/P molar ratio and a lower supersaturation can accelerate the crystallization of ACP into apatite [35,36,37]. The addition of FGF-2 to the supersaturated CaP solution in the present study increased the Ca/P molar ratio of the layer compared with the layer fabricated in the supersaturated CaP solution containing no FGF-2 [24], which indicates that the FGF-2 content of the supersaturated CaP solution itself affects the stability of the layer, but further study of this phenomenon is needed.

## 3. Experimental Section

### 3.1. Preparation of Supersaturated CaP Solutions

Supersaturated CaP solutions were prepared by mixing clinically available infusion and injection fluids. Ca solution (Ca^2+^: 8.92 mM) was prepared from Ringer’s solution (Ca^2+^: 2.25 mM, Otsuka Pharmaceuticals Co., Ltd., Tokyo, Japan) and Calcium Chloride Corrective Injection 1 mEq/mL (Ca^2+^: 500 mM, Otsuka Pharmaceuticals). P solution (PO_4_^3−^: 2.97 mM) was prepared from Klinisalz^®^ (PO_4_^3−^: 10 mM, I’rom Pharmaceuticals Co., Ltd., Tokyo, Japan) and Dipotassium Phosphate Corrective Injection 1 mEq/mL (PO_4_^3−^: 500 mM, Otsuka Pharmaceuticals). FGF-2 solution (100 µg/mL) was prepared by dissolving FGF-2 (Fiblast^®^, Kaken Pharmaceutical Co., Ltd., Tokyo, Japan) in the Ca solution. Meylon^®^ (NaHCO_3_: 833 mM, Otsuka Pharmaceuticals) was used as an alkalizer. From these four solutions, three immersion solutions supplemented with 0.5, 1.0, or 2.0 µg/mL FGF-2 were prepared to immerse Ti pins at 25 °C for 1 day.

Another supersaturated CaP solution containing 4.0 µg/mL FGF-2 was prepared in the same manner as previously reported [17] to immerse Ti pins at 37 °C for 2 days. The FGF-2 concentration in this immersion solution was 4 µg/mL, as in our previous sample (sample F4 in [17]). Instead of Meylon^®^, Bifil^®^ (NaHCO_3_: 166 mM, Ajinomoto Pharmaceuticals Co., Ltd., Tokyo, Japan) was used as an alkalizer, following our previous protocol.

The preparation of the immersion solutions was carried out under sterile conditions. The chemical compositions of these CaP solutions are summarized in Table 1.

**Table 1:** Chemical compositions of the supersaturated CaP solutions used to prepare the 25F0.5, 25F1.0, 25F2.0, and 37F4.0 group samples

Components	Immersion Solution (25F0.5, 25F1.0, and 25F2.0)	Immersion Solution (37F4.0)
	mM	mM
Na^+^	147.23	138.87
K^+^	9.92	7.39
Ca^2+^	8.92	3.67
Mg^2+^	0.24	0.22
Cl^−^	153.46	134.39
H_2_PO_4_^−^	0.95	0.90
HPO_4_^2−^	2.02	0.94
HCO_3_^−^	15.09	15.09
CH_3_COO^−^	1.9	1.80
xylitol	31.65	29.93

### 3.2. Immersion of Ti Pins in the Supersaturated Solution

The Ti pins were commercially available, gamma ray-sterilized, titanium 4.0 mm diameter, 30 mm length [17,21] cancellous screws (Ti pin, #407-030; Synthes Inc., West Chester, PA, USA) with an anodically oxidized surface. Each Ti pin was immersed in 10 mL of an infusion fluid-based supersaturated CaP solution containing 0.5, 1.0, or 2.0 µg/mL FGF-2 at 25 °C for 24 h (25F0.5, 25F1.0, and 25F2.0) or containing 4.0 µg/mL FGF-2 at 37 °C for 48 h (37F4.0), followed by two rinses in 2 mL of distilled injection water (Wasser “Fuso”; Fuso Pharmaceuticals Industries, Osaka, Japan). Subsets of rinsed Ti pins were freeze-dried for later characterization of the composite layer using SEM, energy dispersive X-ray microanalysis (EDX), and powder XRD. The other rinsed Ti pins were used immediately after rinsing without drying for the *in vitro* and *in vivo* experiments.

### 3.3. Characterization of the Composite Layers

The surfaces of the Ti pins were observed by SEM (XL30; FEI Company Ltd., Tokyo, Japan) and EDX (Genesis 2000; EDAX Japan K.K., Tokyo, Japan). The Ti pins were coated with a thin carbon film before observation. To identify the crystalline phase of the surface layer, the layers were scraped off the Ti pin and mounted on a silicon-zero-background plate for analysis using XRD (Rint 2250; Rigaku, Tokyo, Japan). The strength of adhesion between the composite layer and the Ti pin was qualitatively analyzed by a Scotch^®^ tape detachment test as previously reported [38]. A small piece of Scotch^®^ tape was affixed and then detached from the coated pin. The resulting surface of the Ti pin was observed using SEM.

To extract the FGF-2 from the composite layer, the Ti pins coated with the composite layer were immersed in 2 mL of 10 mM citric acid-sodium citrate buffer (pH 5.43 at 25 °C, sterilized) for 3 h to completely dissolve the composite layer. Eight FGF-2 extract solutions were used to determine the amounts of immobilized FGF-2, deposited Ca and P on the surface of the Ti pins, and the mitogenic activity of FGF-2. The amount of immobilized FGF-2 was determined by the Bradford method using a protein quantitation kit (Bio-Rad Laboratories Inc., Hercules, CA, USA). The amounts of deposited Ca and P were determined using an ICP (SPS7800, Seiko Instruments Inc., Chiba, Japan). 

The mitogenic activity of the FGF-2 in the composite layers was evaluated using the proliferation of NIH3T3 cells as described in a previous study [20]. Briefly, an FGF-2 extract (50 µL) was added to 1.0 mL of serum free Dulbecco’s modified Eagle’s medium supplemented with l-glutamine (0.3 mg/mL), bovine serum albumin (1.0 mg/mL), insulin (5.0 µg/mL), and transferrin (1.0 µg/mL) in a well of a 24-well plate in which 1 × 10^4^ fibroblastic NIH3T3 cells (NIH3T3-3-4, RIKEN BioResource Center, Ibaraki, Japan) had been precultured for 1 h. After another 72 h of culture, the number of NIH3T3 cells was counted using a cell counting kit (CCK-8 kit, Dojindo Laboratories, Kumamoto, Japan).

### 3.4. Animal Experiments

The surgical technique used was the same as that described in our previous studies [17,21,24]. Ti pins were implanted into 60 skeletally mature male Japanese white rabbits weighing approximately 3.0 kg by a single physician who was blinded to the pin identifications. The rabbits were divided into four groups with 10 animals each: 25F0.5, 25F1.0, 37F4.0, and the uncoated Ti pin (UN1) group. Similarly, Ti pins in the 25F2.0 and another uncoated Ti pin (UN2) groups were implanted by the same physician in 10 rabbits each as an additional experiment. Percutaneous implantation of the Ti pins in the rabbit proximal tibial metaphyses (Figure 7) was performed following previously described methods [17,21,24]. Briefly, small (10 mm) incisions were made aseptically in the skin at the medial proximal tibia after an intravenous injection of barbiturate (40 mg/kg body weight). The skin and subcutaneous tissue were separated from the periosteum. Second incisions were made on the periosteum. The periosteum was elevated and dissected from the underlying tibia. Next, a 2.5-mm diameter hole was drilled into the tibial metaphysis with individual taps for each pin. The Ti pins were then manually inserted into these holes. Two Ti pins from the same group were implanted individually in bilateral proximal tibial metaphyses of each rabbit. Hence, 20 Ti pins were implanted for each group. After the implantation, the skin was tied with two 3-0 nylon sutures (SP17G03N-45; BEAR Medic Corporation, Tokyo, Japan) bilaterally at the screw. Postoperatively, each rabbit was allowed free activity in its cage. The rabbits did not receive any antibiotics or treatment for their wounds, or any postoperative analgesics. All of the rabbits were sacrificed by an intravenous overdose administration of pentobarbital 4 weeks after the operation.

All animal experiments and breeding were performed according to the conditions approved by the ethics committees of both the University of Tsukuba and the National Institute of Advanced Industrial Science and Technology (AIST). All activities were implemented in accordance with the National Institutes of Health Guidelines for the Care and Use of Laboratory Animals [39].

### 3.5. Classification of Pin Tract Infections by Visual Inspection

Four weeks after implantation, the presence of pin tract infections was evaluated using a modified Checketts classification before sacrifice [17]. Grade 0v corresponds to “no redness”, in which no redness, discharge, or pin loosening was observed. In other words, “no redness” means no infection in both the soft and bone tissues. Grade 1v corresponds to infections only in the soft tissue, characterized by redness and discharge around the pin, but without pin loosening. Grade 2v corresponds to infections in both the soft and bone tissues, characterized by redness and discharge around the pin with associated pin loosening caused by osteomyelitis. A single physician who was blinded to the group identification of the pins evaluated the rabbits for pin tract infections.

### 3.6. Histological Analysis

After collection of the exudate from the pin tracts, the proximal tibial metaphyses were fixed in 10% neutral buffered formalin for 7 days and then separated into the soft- and hard-tissue parts. The soft-tissue parts were undecalcified and embedded in paraffin, and the hard-tissue parts were decalcified in an ethylenediaminetetraacetic acid solution and embedded in paraffin. Sample sections were cut 5 μm thick, perpendicular to the tibial longitudinal axis, and parallel to the hole of the pin. Sections were stained with hematoxylin-eosin. The specimens were observed histologically using a light microscope (BX-51; Olympus Optical Co., Ltd., Tokyo, Japan) to evaluate the grade of pin tract inflammation in the soft and bone tissues.

For the soft tissue, Grade 0s corresponds to “no inflammation”, in which no inflammation was observed in the surrounding soft tissue along the entire length of the boundary between the pin and soft tissue. Grade 2s corresponds to “severe inflammation,” in which inflammation was observed in the surrounding soft tissue along the entire length of the boundary between the pin and soft tissue [21,24]. Grade 1s is a status between Grades 0s and 2s, corresponding to “partial inflammation,” in which inflammation was observed in the surrounding soft tissue along only part of the length of boundary between the pin and soft tissue.

Similarly for the bone tissue, Grade 0b corresponds to “no osteomyelitis,” in which no inflammation was observed in the surrounding bone tissue along the entire length of the boundary between the pin and bone tissue. Grade 2b corresponds to “severe osteomyelitis,” in which inflammation was observed in the surrounding bone tissue along the entire length of the boundary between the pin and bone tissue [24]. Grade 1b is a status between Grades 0b and 2b, corresponding to “partial osteomyelitis,” in which inflammation was observed in the surrounding bone tissue along only a part of the length of the boundary between the pin and bone tissue.

A single physician who was blinded to the pin identifications evaluated the soft and bone tissues for pin track inflammation.

### 3.7. Bacterial Culture and Identification

After complete removal of the Ti pins, the exudate around each pin was collected with a cotton swab. Those swabs were delivered to a company for clinical laboratory testing (SRL Inc., Tachikawa, Tokyo, Japan) of the major bacterial species present: *S*. *aureus* and *E*. *coli* [24].

### 3.8. Biomechanical Analysis

After sacrificing the rabbits, the peak extraction torque for each Ti pin was measured using a mechanical torque-measuring system (HTG2-5N; Imada Co., Ltd., Toyohashi, Japan) [17,24].

### 3.9. Statistical Analyses

The amount of Ca and P, Ca/P molar ratio, and FGF-2 content for the four groups (25F0.5, 25F1.0, 25F2.0, and 37F4.0) were compared using a one-way analysis of variance with Tukey-Kramer *post-hoc* tests. The mitogenic activities of each group were compared using Student’s *t*-tests. The results of the pin tract infections by visual inspection and histological analyses in each group were analyzed by χ^2^ test for independence. The bacterial detection data from each group were analyzed by χ^2^ test for independence. The extraction torque data from each group were compared using Student’s *t*-test. The significance level was set at *p* < 0.05 for each analysis.

## 4. Conclusions

FGF-2-apatite composite layers were successfully fabricated on Ti pins at 25 °C for 1 day in one step, as well as at 37 °C for 2 days. The results of *in vitro* assays confirmed that the FGF-2 immobilized in the composite layers retained its mitogenic activity. The FGF-2-apatite composite layer formed at 37 °C reduced the pin tract infection rate and increased the fixation strength at the bone-pin interface. Moreover, this composite layer reduced the invasion rate of *S. aureus* into the pin tract. On the other hand, the FGF-2-apatite composite layers formed at 25 °C were less effective at both improving the fixation strength at the bone-pin interface and at resisting pin tract infections. This reduced effectiveness may be attributed to the lower Ca/P molar ratio and resulting chemical instability of the composite layer, or a change in optimum FGF-2 content due to the change in Ca/P molar ratio. Further optimization of the fabrication parameters is needed to reduce the bacterial infection rate and improve the fixation strength at the bone-pin interface for the FGF-2-apatite composite layers prepared at 25 °C. The results of this study suggest that the chemistry of the calcium phosphate matrix that embeds FGF-2, in addition to FGF-2 content and activity, has a significant impact on composite infection resistance and fixation strength.
